# An Essay Towards the Improvement of Some of the Important Instruments in Surgery; and of the Operations in Which They Are Employed

**Published:** 1816-11

**Authors:** 


*An Essay towards the Improvement of some of the important Instruments in Surgery; and of the Operations in which they are employed.* By WILLIAM JARDINE, Surgeon, R. N. Edin. pp. 180. With numerous plates.

Mr. JARDINE appears to have a very ingenious turn for mechanics, and we regret that we are unable, by verbal description, to do justice to the many improvements which, we really think, he has suggested in the construction of instruments, and in the manner of applying them. We shall glance hastily at some of Mr. Jardine's proposals in the order of their standing in his book.

The *Raspatory.* That in common use being triangular, he conceives it very unfit for its purpose, and therefore proposes an instrument somewhat resembling a cooper's marking iron; which, turning circularly, like a pair of compasses, removes no more than is necessary of the pericranium. This instrument, if used with the saw, will greatly accelerate the operation, as it will perforate the cranium much sooner than can be done with the saw. It may be adjusted to saws of different diameters, by means of a slide, to which the blade in use is fixed.

*Trephine.* The improvement here is to prevent the instrument from slipping suddenly in upon the brain. It can be used as safely at sea as on shore.

"The most timid operator cannot, with such an instrument, be under the least apprehension of plunging in the trephine as he formerly was."

It is shaped like a common trephine, with two rims on its crown or barrel, the one above the other. The upper rim is screwed on, the under moves easily a little up and down upon the crown. They are separated by a spiral ring round the crown affixed to both rims, and which admits of their approaching each other when some pressure is made, but evidently prevents a sudden jerk.

The cutter and saw for removing angular and other irregular portions of bone would probably prove more effectual than Hey's saw; but of this we cannot speak with certainty, as theory and practice are often at such variance.

Mr. Jardine conceives that the common trephine may be greatly improved by being divided into several portions, any one of which may be depressed by means of a screw. This will obviate the necessity the operator is frequently under of inclining the trephine to one side. This improved trephine will cut openings of any different size by depressing a division of the circle and shifting the centre.

"If unequally divided, and the parts taken separately, it will furnish a curved saw of different segments on the same radius."

*Elevator.* The instrument in use at present often gives great trouble to the operator, in attempting to raise the depressed portions of a bone, particularly of the os frontis. They are sometimes found to be totally inadequate to the desired purpose. Mr. Jardine proposes, in different cases, "to screw the elevator through the top of a bipes or trivet."

"The prop may be fitted to the unequal convexity of the head by lengthening or shortening the feet."

The dura mater may be detached from the sawn piece of bone by a piece of wire, nearly the same thickness as the plate of the saw, "bent about half an inch at one end into a right angle, and then horizontally curved, so that it may be let into the cut or circle of which that curve is a part, and turned so as to separate, as far as it can reach the membrane from the bone."

The *Lenticular.* In the Medical Journals of London for December 1812, is a description by Mr. Jardine of a lenticular for smoothing the edges of a round perforation of the cranium. He now proposes another form of that instrument, with which the edges of an opening of any form may be easily smoothed.

"It is made of a rod of iron, about six or seven inches long, bent nearly into the form of a pair of compasses. It is widened by its spring. The blade is about the tenth of an inch from the points, which are bent outwardly that length, to keep the instrument down in its place when in the perforation." "By turning this instrument in a round perforation, the blades are pressed by the spring against the sides of the opening, and take off at once all the ragged and splintered parts. In an oblong opening, the perforation must be gradually smoothed, by working one blade a little forward on one side, and the other on the opposite side till they are brought to a point. In withdrawing it, the sides are compressed, as when it is introduced."

He recommends that the splinters, that are too minute for the forceps, be taken up with the end of a small linen roller dipt into some mild ointment to which they will adhere.

*Instrument for extracting Teeth.* The instrument Mr. Jardine has directed to be made, has the prop of the claw, when set, not vertical, as in the common instrument, but in an arch of forty-five degrees from the vertical point. The tooth being, by this instrument, raised in the ascent of an arch, "the operation will become easier and safer, and the tooth not so likely to be broken, or the jaw so often splintered, as it is in the usual way.

*Instrument for extracting Fish Bones, Pins, &c. sticking in the Œsophagus.* As many sharp-pointed substances cannot, without danger of wounding the œsophagus, be forced into the stomach, Mr. Jardine proposes an instrument for extracting such substances, which seems peculiarly applicable to cases in which medical assistance cannot be immediately obtained, as it is so easy in the application, that any one to whom its principles have been once explained need not hesitate to apply it.

"It is made of five or six threads of catgut, nearly three inches long, twisted round the end of a wire that passes through a flexible pipe, and protrudes about two inches and a half beyond its extremity; one end of the twist is fixed to the end of the pipe, and the other to the end of the wire. In the extended form, it is to be introduced so far into the throat, that all the catgut may be supposed to have got beyond the bone or thing to be extracted. The ring at the end of the handle, and outside of the mouth, is then to be drawn out about an inch to spread the catgut, and in withdrawing the instrument the bone will be extracted with it."

*Fistula.* On account of the difficulties that occur in performing the operation for fistula in ano, Mr. Jardine proposes to make the incision "by running a scalpel with a fistular back upon a wire or probe previously passed through the sinus, and drawing it down a canula, which it meets in the return, or by drawing down both together." By this method, the operation may be performed higher in the rectum than the finger can reach.

*Of sawing off the Ends of diseased or fractured Bones.*— One objection to this operation is the size of the wound, which is unavoidably extensive, in the present method of cutting the bone with the common amputation saw. It is proposed, therefore, to perform this operation by a circuitous saw, shown in Plate X. Figure 1. The blades pass one another like a pair of scissars, cutting however close enough to divide the bone. There is also an instrument to support the blades, and bear off the integuments defending them from the friction of the saw during the operation. Mr. Jardine is of opinion, that, "with a trephine large enough, the bone might be cut, in this operation, as soon as with any thing; although it would make two cuts instead of one, it would, nevertheless, complete the division much sooner than is imagined, from the confidence with which the operator would work it in comparison with what he would do when perforating the cranium."

*Bandages.* Mr. Jardine justly observes, that "it is very difficult to describe the form of a bandage, or direct how it is to be applied, so as to be easily understood." He therefore recommends to the young surgeon practice on a bust, or something like the part to which the bandage is to be applied; and has, from his own experience, suggested several useful and simple modes of applying bandages and splints. There is a greater variety in dressing than in operating, and in some cases more dexterity is required in the former than in the latter; indeed, in many operations and wounds, the cure depends more upon the management of the dressings afterwards, than upon the operation itself.

"In some very obstinate and troublesome cases, one improved dressing, according to my own observation, has made the most sensible difference, both in the appearance of the wound, and the quantity and quality of the discharge."

He imputes the prejudices of some surgeons against the use of splints in fractures, to the unfitness of splints for their purpose, or to their being tightened too soon. All that is required of splints, before the swelling and inflammation subside, is to preserve the steadiness of the limb against the necessary and inadvertent motion of the body. As the swelling subsides, and the callus hardens, the splints should be gradually tightened, as the adjustment of the limb then requires more particular attention.

"If we are to judge from analogy, gentle pressure should have some effect in shaping the callus, while it is compressible, as well as the other soft parts, which we know it is so effectual in, as in the shaping of a stump, or checking the excrescent parts of a wound, &c."

The most convenient splints he considers to be those made of whalebone or some elastic wood, incased in canvass or coarse linen, in the same way in which whalebone is covered in womens' stays. The cases are open at each end, and considerably longer than the bones they contain, that by them the splint, when too short, may be lengthened to the size of the limb, merely by thrusting every other splint or bone forward by a wire, almost thick enough to fill the place occupied by the bone. When too wide, a proportionate number of bones is to be drawn out of one or both sides, to fit it to the breadth required.

"These splints may, in particular cases, be laid on the tibia without any inconvenience whatever, provided two or three of the bones above it are drawn out."

Our limits forbid us proceeding farther; but we hope that the above specimens will be sufficient to excite the attention of practical operators and surgical instrument-makers to the subject of Mr. Jardine's book, which contains very many judicious observations on several practical points of surgery which will repay the perusal.

---

